# A Study on Community Formation Patterns Based on the Usage of Benches by Seniors

**DOI:** 10.1093/geroni/igaa057.1411

**Published:** 2020-12-16

**Authors:** Yunjeong Lee, Akiko Nishino, Yuji Matsuda, Toshio Otsuki, Kazuhiko Nishide

**Affiliations:** 1 ChungNam Institute, Chungcheongnam-do, Republic of Korea; 2 The University of Tokyo, Tokyo, Japan

## Abstract

In Japan, the rate of aging exceeds 27%, and the decline of communities has been an issue. Therefore, in this study we take the T housing complex as an example where the rate of aging is more than 40% and aim to understand the usage pattern of benches. An observational research was conducted for 19 days to examine the usage of a total of 23 benches, 17 existing ones and 6 newly installed ones, in the T housing complex. This investigation led to the findings. An average of 417 people used the benches in a day. Research determined that there are four different types of bench utilization, and they each present different ways of building relationships with other people. Bench use behavior was broken into four different categories. Sitting on a bench alone (without building relationships with others) was classified as “single use”. Sitting on a bench with another person but not engaging in any relationship-building behavior was classified as “sharing”. Sitting on a bench and exhibiting relationship-building behavior with others (such as through talking) was classified as “chatting”. Sitting with companions in groups of 3 or more people and exhibiting relationship-building behavior was classified as “group use”. Our findings provide evidence that a bench can be a device to foster the formation of diverse communities.

